# Supplementary material for: Molecular mechanism of hyperactivation conferred by a truncation of TRPA1
Source: Nat Commun. 2023 May 19;14:2867. doi: 10.1038/s41467-023-38542-1 (PMC10199097; doi:10.1038/s41467-023-38542-1)
Supplement: Supplementary file 1 — Supplementary Information [file 41467_2023_38542_MOESM1_ESM.pdf]

Supplementary Information

Supplementary Methods

Plasmid Construction and Molecular Biology

DNA sequences for human TRPA1, human TRPV1, and rat TRPM8 were amplified by PCR and subcloned into p3xFLAG-eYFP-CMV-7.1 vector (Addgene #34582) at the NotI/BamHI sites or into 8xHis-MBP pFastBac1 modified with a CMV promoter (obtained from David Julius) at the BamHI/NotI sites using In-Fusion EcoDry cloning (Takara) according to manufacturer protocols. Mouse TRPA1 and zebrafish TRPA1a orthologs were similarly subcloned into p3xFLAG-eYFP-CMV-7.1.

For quantifying non-functional TRPA1 constructs by ratiometric calcium imaging, 3xFLAG-WT or R919* hTRPA1 were subcloned into the pIRES2-eGFP vector (Addgene #111499) at XhoI/EcoRI sites using In-Fusion EcoDry cloning according to the manufacturer protocols.

For tandem purification studies, a Strep-tag II (Trp-Ser-His-Pro-Gln-Phe-Glu-Lys) or a FLAG tag (Asp-Tyr-Lys-Asp-Asp-Asp-Asp-Lys) was added on the N-terminus of MBP WT or R919* TRPA1 using Quikchange Lightning site-directed mutagenesis (Agilent).

8xHis-BioID2-tagged variants were generated by introducing a BamHI site between the 8xHis and MBP tags in the 8xHis-MBP-pFastBac1 modified with a CMV promoter vector by Quikchange Lightning site-directed mutagenesis (Agilent) and subcloning BioID2 into the BamHI sites using In-Fusion EcoDry cloning according to manufacturer protocols.

For dual-color FSEC experiments, WT hTRPA1 was subcloned into the pEG BacMam N-term His8 GFP vector and R919* hTRPA1 was subcloned into the pEG BacMaM N term StrepII eGFP 3C vector (Addgene). In the latter, the StrepII tag was changed to a FLAG-tag by site-directed mutagenesis. To generate FLAG-mVenus-R919* hTRPA1, the following mutations were introduced into eGFP using site-directed mutagenesis: F47L, S73G, T66G, V69L, M154T, V164A, S176G, and T204Y. To generate 8xHis-mCerulean-WT hTRPA1, mCerulean3-C1 (Addgene) was used to generate an mCerulean megaprimer to replace eGFP by site-directed mutagenesis.

All DNA primers were ordered from ThermoFisher, and all constructs were sequence-verified using the Yale School of Medicine Keck DNA Sequencing Facility. Details for all plasmids and primers used to develop constructs used in main manuscript figures are included in the accompanying Source Data file.

Ratiometric Calcium Imaging

AITC, Carvacrol, Capsaicin, Menthol, and Cinnamaldehyde were all purchased from Sigma and were freshly prepared as stocks at 4x the desired concentration in 1% DMSO and Ringer's solution. 5 µL 4x agonist was added to wells containing 15 µL Ringer's solution to give the final 1x desired concentration. For the antagonist experiments in Fig. S3, cells were pre-treated with Ringer's solution containing A-967079 (Tocris), HC-030031 (Tocris), Ruthenium red (Sigma), or Carbamazepine (Sigma) at the desired concentration for 1 min and activated with a 4x AITC solution containing 4x antagonist to maintain antagonist concentration. When quantifying calcium influx responses for dose-response curves, each experimental replicate at each agonist concentration was an average response from 30 cells, with three separate replicates (*e.g.*, 90 total cells per agonist concentration). For all other experiments, a minimum of 60-90 cells were selected per condition per replicate for ratiometric fluorescence quantification in Metafluor v7.8.13 with 3-5 replicates per experiment. Pre-active cells were excluded from quantification and background signal was quantified from un-transfected cells and subtracted from quantified cells for normalization. Unless reported in arbitrary units (arb. units), all data were normalized by the maximum response evoked for that transfection condition by 10x agonist (100 uM for human and mouse TRPA1, and 1 mM for zebrafish TRPA1), 200x agonist (200 µM Menthol), or 1000x agonist (1 µM Capsaicin). When validation of equivalent expression was required, the imaged cells were lysed, and quantitatively immunoblotted for 3xFLAG-tagged TRPA1 as detailed below. For inactive variants or antagonist-treated TRPA1 that did not exhibit appreciable calcium influx, constructs in the pIRES-eGFP vector were

transfected in HEK293T cells. Fluorescence images were collected with a GFP filter after ratiometric calcium imaging, which allowed selection of GFP positive cells for subsequent quantification. R919* hTRPA1 data was quantified as above.

Immunofluorescence Imaging
HEK293T cells transiently transfected using Lipofectamine 2000 (ThermoFisher) according to manufacturer's protocol were incubated for 20 hours prior to immunostaining. Cells were fixed on coverslips with 3.5% paraformaldehyde for 15 minutes, and then incubated with 5 ug/mL Alexa Fluor 350-conjugated Wheat Germ Agglutinin (Invitrogen) in PBS at room temperature for 10 minutes, followed by permeabilization using PBS + 0.5% Triton X-100 for 10 minutes and incubation with PBS + 1% Bovine serum albumin (BSA) for 10 minutes. Cells were incubated with primary antibodies (anti-FLAG, mouse, Sigma F1804; anti-GFP, rabbit, Invitrogen G10362) diluted 1:1000 in PBS + 1% BSA for 16 hours at 4ºC. Cells were washed with PBS and incubated with secondary antibodies (AffiniPure goat anti-mouse IgG AlexaFluor594-conjugated, Jackson ImmunoResearch 115-585; AffiniPure goat anti-rabbit IgG AlexaFluor488-conjugated, Jackson ImmunoResearch 111-545) diluted 1:1000 in PBS + 1% BSA for 1 hour at room temperature. Images were acquired on a Nikon ECLIPSE Ti2 with a Hamamatsu Fusion sCMOS camera at 100X magnification.

Image Analysis and Deconvolution
For quantitative analysis of R919* TRPA1 intensity at the plasma membrane, raw single stack immunofluorescence images were processed in ImageJ using the line scanning function. A straight line was drawn through the width of at least 30 cells and the fluorescence intensity of each pixel was measured as a function of distance for each channel. Intensity was normalized to the average middle 30% of each line scan representing the cell interior, and distance was normalized from 0 to 1 to account for differences in cellular size. Line scans were compiled using GraphPad Prism, and the average maxima from all scans was taken to represent fluorescence intensity at the plasma membrane. For quantitative analysis of WT and R919* TRPA1 co-localization, raw single stack immunofluorescence images were processed in ImageJ using the line scanning function. Freehand lines were drawn over the plasma membrane, and the intensity of at least 450 pixels were measured for each channel. Measurements were normalized to the average intensity of each line scan to account for variability in transfection efficiency. Pearson's correlation coefficient (r) and the coefficients of determination (r^2) were calculated to represent WT and R919* TRPA1 colocalization. To deconvolve immunofluorescence images, stacks of optical sections (z step = 0.2 μm) were restored with Huygens software (Scientific Volume Imaging) using the maximum-likelihood estimation algorithm. The restored stacks were further processed in Photoshop CS4 (Adobe).

Biotinylation Assays
Surface biotinylation assays were adapted from previously reported protocols [1]. Briefly, HEK293T cells were seeded in a 6-well plate pre-coated with Poly-L-Lysine (Sigma) and transfected with expression plasmids. 40-48 hours post-transfection, cells were washed with PBS, placed on ice, and incubated for 20 minutes with chilled 0.05 mg/mL Sulfo-NHS-LC-Biotin (Thermo Fisher) in Ringer's solution. Cells were then washed with chilled wash buffer (PBS supplemented with 100 mM Glycine) and the reaction was quenched on ice for 30 minutes with 100 mM Glycine and 0.5% BSA in PBS. Cells were then washed three times and lysed in TRPA1 lysis buffer (50 mM HEPES, 150 mM NaCl, 1 mM IP_6, 20 mM DDM, 1x EDTA-free Roche Complete Protease Inhibitor Cocktail, pH 7.8) supplemented with 100 mM Glycine directly on the 6-well plate. Lysates were collected, protein concentration was determined by BCA assay (Pierce), biotinylated proteins were isolated by Neutravidin pulldown and analyzed by immunoblot as described below. During quantification, any FLAG signal in the torsin eluates lane was subtracted from all other conditions to account for probe internalization.

For proximity biotinylation assays, HEK293T cells co-expressing 8xHis-BioID2- or 3x-FLAG-tagged TRPA1 and Kv1.2/2.1 fusion proteins were treated with 50 uM biotin supplemented into culture media ~24 hours post-transfection. Cells were lysed 16 hours later with TRPA1 lysis buffer and affinity purified as below.

Pulldown Experiments
40-48 hours post-transfection, HEK293T cells were lysed in TRPA1 lysis buffer. Total protein concentration was quantitated using BCA assay (Pierce). Equal concentrations of protein lysate from each experimental

condition were added to affinity resins. 10% of loaded protein amount was reserved as a whole-cell lysate loading control. Affinity purification, PAGE, and immunoblotting were performed as described below.

FLAG Immunoprecipitation: Lysates were incubated with EZview Red Anti-FLAG M2 affinity resin (Sigma) for 1 hour at 4°C with gentle rotation. Resin beads were washed four times with lysis buffer prior to elution with 125 µg/ml 3xFLAG peptide (Sigma).
Amylose Pulldown: Lysates were incubated with amylose resin (New England Biolabs) for 1 hour at 4°C with gentle rotation. The resin beads were washed five to six times with lysis buffer prior to elution in 60 mM maltose (Sigma).

Strep Pulldown: Lysates were incubated with Strep Tactin Sepharose (IBA) for 1 hour at 4°C with gentle rotation. The resin beads were washed six times with lysis buffer prior to elution in 1X Buffer E (IBA). For tandem pulldown assays, Strep-enriched eluates were subsequently incubated with EZview Red Anti-FLAG M2 affinity resin for 2 hours at 4°C with gentle rotation and washed 6 times with lysis buffer prior to elution as above.

Neutravidin Pulldown: Cell lysates that were generated following surface labeling or proximity biotinylation experiments were incubated with Neutravidin resin (Pierce) for 2 hours at 4°C with gentle rotation. The resin was then washed with lysis buffer three times, followed by a harsher wash with 1x PBS supplemented with 100 mM DTT. Resin was then washed once each with lysis buffer and 1x PBS. Biotinylated protein was eluted from the resin with a multi-step protocol to prevent TRPA1 aggregation while maximizing protein elution from the resin. First, resin was incubated with 10 µL of biotin elution buffer (TRPA1 lysis buffer, 100 mM Glycine, 10 mM Biotin, 1% SDS) on ice for 10 min, followed by addition of 1 µL β-mercaptoethanol (BME; Sigma) to each sample and incubation on ice for 5 minutes, and finally by addition of 4 µL 4x Laemmli buffer supplemented with 10% BME and incubated at 65°C for 10 minutes. The resin was centrifuged, supernatant was removed and combined with additional 4 µL Laemmli buffer supplemented with 10% BME for SDS-PAGE analysis.

Ni-NTA pulldown: Cell lysate samples were incubated with His-Pur Ni-NTA resin (ThermoFisher) for 30 minutes at 4°C with gentle rotation. Resin was washed 3x with lysis buffer and eluted with 400 mM imidazole.

SDS-PAGE and Immunoblot
Samples were combined with 4x Laemmli Sample buffer supplemented with 10% BME, separated on pre-cast 4-20% SDS-PAGE gels (BioRad), and transferred onto PVDF membranes (BioRad) by semi-dry transfer. Blots were blocked in 3% BSA prior to antibody probing. The following primary antibodies were used in 1x PBST buffer (Boston Bioproducts): MBP (mouse, 1:30,000, New England Biolabs E8032), FLAG (mouse, 1:30,000, Sigma A8592), strep (mouse, 1:30,000, IBA 2-1509-001), tubulin (mouse, 1:5,000 in BSA, Sigma T6199), HA (rat, 1:2,000 in BSA, Roche 12013819001), and His probe (1:50,000, Thermo 15165). HRP-conjugated IgG secondary anti-mouse antibody was used as needed (rabbit, 1:25,000, Thermo 61-6520). Membranes were developed using Clarity Western ECL substrate (Bio-Rad) and imaged using a Chemidoc Imaging System (BioRad). Densitometric quantifications were performed with ImageJ software. All quantified band intensities for eluted samples were normalized to their tubulin-normalized input band intensities, except for the tandem purification crude stoichiometric analysis in Fig. 5f where only MBP signal intensity in eluates was used. All uncropped Western blots from experiments throughout the manuscript are presented in Supplementary Figures 14-17.

Intracellular ROS detection
Intracellular generation of ROS was measured via conversion of 2,7 dihydro-dichlorofluorescein diacetate (H_2DCF-DA) into the fluorescent product dichlorofluorescein (DCF) upon oxidation. Our procedure was adapted from previously reported protocols [2]. Briefly, HEK293T cells transfected with empty vector (mock) or hTRPA1 variants were seeded into a 96-well dish 20 hours post-transfection. 40 hours after transfection, cells were loaded with 50 µM H_2DCF-DA dye in Ringer's solution for 1 hour at 37 °C. Cells were then washed 1x and resuspended in Ringer's solution. Fluorescence was measured in a BioTek SynergyMx plate reader (excitation: 488 nm ; emission 525 nm). Background readings were from wells containing Ringer's solution only. H_2DCF-DA dye treated with 400 µM H_2O_2 was the positive control.

ER Stress Assay

C-terminally truncated Lamin B receptor (LBR1600*) has been shown to be metabolically unstable, and generally undergoes rapid turnover that is partially dependent on ERAD factors such as p97, Ufd1, Npl4, and Hrd1 [3,4]. When these factors are pharmacologically inhibited, LBR1600* is stabilized, and can therefore be used as a model substrate for ERAD. Our procedure was adapted from previously reported protocols [4]. Briefly, HA-tagged LBR1600* was co-transfected into HEK293T cells alongside empty vector (mock) or 3xFLAG-WT and 3xFLAG R919* hTRPA1. 20-40 hours post-transfection, cells were treated with 10 μM of p97 inhibitor CB5983 in Ringer's solution for 0, 4 or 8 hrs. Cells were then lysed, analyzed by SDS-PAGE and Western blot, as described above.

Fluorescence Size Exclusion Chromatography (FSEC)

FSEC procedures were adapted from those previously reported [5]. Briefly, sample volumes were brought to 1 mL with TRPA1 lysis buffer, then passed through 0.22-micron filters (Costar) by centrifugation, loaded onto a Superose 6 Increase column (GE Healthcare) pre-equilibrated with FSEC buffer (20 mM HEPES, 150 mM NaCl, 1 mM BME, 0.5 mM DDM, 1 mM IP_6, pH 8), and run at a flow rate of 0.5 ml/min. The in-line fluorescence detector (Shimadzu) spectral settings were as follows: Ex:488; Em: 510 nm (eGFP), Ex:515; Em:528 nm (mVenus), Ex: 433; Em: 475 nm (mCerulean); time increment, 1 s; integration time, 1 s. Data were collected with Unicorn v7.2 software. Fractions corresponding to TRPA1 and free FLAG peptide peaks were collected, concentrated using Amicon Ultra 10 KDa cut-off spin filters, and subjected to immunoblot analysis.

For experiments in Fig. 5c, GFP- and 3xFLAG-tagged constructs were co-expressed in the indicated combinations for 40-48 hours. Lysates were subjected to FLAG immunoprecipitation prior to FSEC analysis.

For dual-color experiments in Supplementary Fig. 7, cells were transfected with 8xHis-mCerulean-WT hTRPA1 and/or FLAG-mVenus-R919* hTRPA1. Cells were lysed 40 hours post-transfection. For Supplementary Fig. 7b, lysates were subjected to Nickel-FLAG tandem purifications. For Supplementary Fig. S7d and e, whole cell lysates were used. Eluates or lysates were divided into two and analyzed sequentially with FSEC, first with mCerulean and second with mVenus fluorescence detection settings. Tandem purified samples were also analyzed by immunoblot analysis in Supplementary Fig. 7c.

Blue Native PAGE

Lysates containing dual-tagged WT and R919* TRPA1 were tandem-purified with strep and FLAG affinity resins as described above. Eluates were split into two aliquots, one of which was given full denaturation treatment in Laemmli Sample buffer supplemented with 10% BME, while the second was added to NativePAGE Sample Buffer and 5% G-250 Sample Additive (Invitrogen) as per manufacturer instructions. Samples were loaded onto 4–15% Mini-Protean TGX Gels (Bio-Rad) and run at 180 V in NativePAGE Running Buffer (Invitrogen) with 10x Coomassie additive in the cathode buffer. After 25 min, the voltage was adjusted to 115 V for the remainder of the run. After 40 min of run time, the 10x Coomassie cathode buffer was removed and replaced with 1x Coomassie running buffer for the remaining 1.5 hours. Transfer to PVDF membrane and immunoblotting were performed as described above.

Supplementary Figures

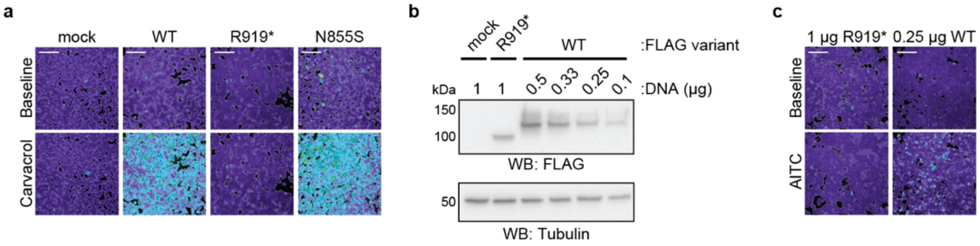

Supplementary Figure 1. The R919* mutant is a nonfunctional TRPA1 natural variant. (a) Ratiometric calcium imaging of HEK293T cells transiently transfected with empty vector (mock), WT hTRPA1, R919* hTRPA1, or N855S hTRPA1. Cells were stimulated with Carvacrol (100 µM). Images are representative of three independent experiments. (b) Western blot of lysates from transiently transfected HEK293T cells expressing 3xFLAG-tagged hTRPA1 variants, probed as in (Fig. 1f). HEK293T cells were transfected with the indicated amount of plasmid (in µg). Blot is representative of three independent experiments. Full blots are included in Supplementary Fig. 16. (c) Ratiometric calcium imaging of HEK293T cells transiently transfected with the indicated amount of 3xFLAG-tagged WT or R919* hTRPA1 to achieve comparable expression levels. Cells were stimulated with AITC (100 µM). Images are representative of three independent experiments. (a and c) Scale bars indicate 100 µm.

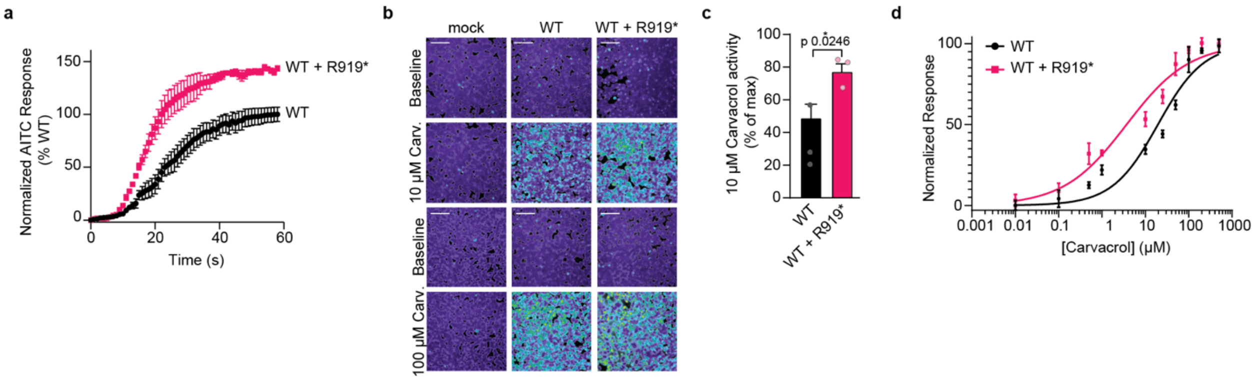

Supplementary Figure 2. The R919* mutant confers hyperactivity when co-expressed with WT TRPA1 subunits. (a) Time-dependent ratiometric calcium imaging responses evoked by 10 µM AITC normalized to maximum response elicited from WT (black) or WT and R919* (pink) hTRPA1-expressing cells. Data represent mean ± SEM. n = 3 independent experiments, n ≥ 90 cells per condition per experiment. (b) Ratiometric calcium imaging of HEK293T cells transiently transfected with empty vector (mock), WT hTRPA1, or WT and R919* hTRPA1. Cells were stimulated with Carvacrol (10 or 100 µM). Images are representatives from three independent experiments. Scale bars indicate 100 µm. (c) Quantification of 10 µM Carvacrol-evoked change in Fura-2 ratio relative to maximum response of each expression condition at 100 µM Carvacrol. Colors as indicated in (a). Data represent mean ± SEM. *p<0.05. n = 4 independent experiments, n ≥ 90 cells per transfection condition per experiment, two-tailed Student's t-test. (d) Dose-response curve of Carvacrol-evoked calcium responses for HEK293T cells transiently transfected with WT hTRPA1 (black) or WT and R919* hTRPA1 (pink). Calcium responses normalized to maximum calcium response to 500 µM Carvacrol. Traces represent the average ± SEM of normalized calcium responses from 3 independent experiments, n = 30 cells per agonist concentration per experiment. Data were fit to a non-linear regression. EC_{50} (95% CI) values are 19.5 µM for WT hTRPA1 (95% CI, 9.6-33.8 µM) and 3.7 µM for WT and R919* hTRPA1 (95% CI, 1.9-6.8 µM).

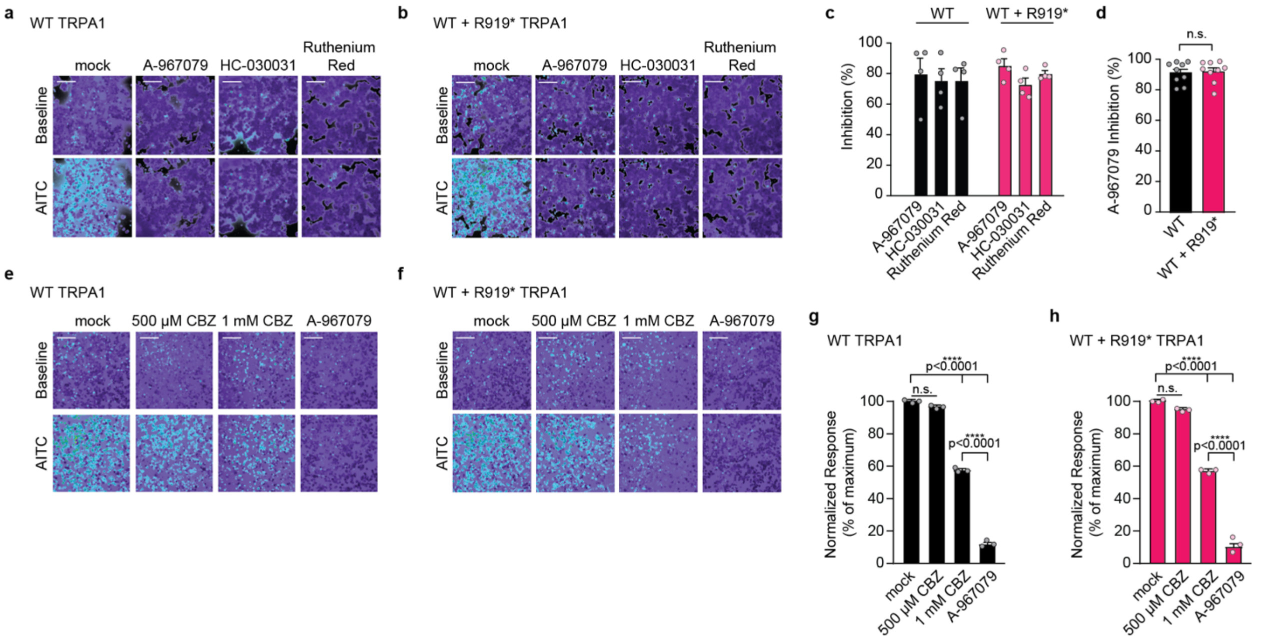

Supplementary Figure 3. WT and WT + R919* channels are inhibited by canonical TRPA1 antagonists and pore blockers. (a-b) Ratiometric calcium imaging of HEK293T cells transiently transfected with (a) WT hTRPA1, or (b) WT and R919* hTRPA1. Cells were pre-treated with Ringer's solution (mock), A-967079 (10 µM), HC-030031 (30 µM), or Ruthenium Red (10 µM). Channels were activated with AITC (100 µM). Images are representatives from four independent experiments. (c) Quantification of antagonist-mediated inhibition of 100 µM AITC-evoked changes in Fura-2 ratio in cells from B and C expressing WT (black) or WT and R919* (pink) hTRPA1. Data represent mean ± SEM of percentage of inhibition of the AITC-evoked maximum response for both transfection types. n = 4 independent experiments, n ≥ 90 cells per condition per experiment. (d) Quantification of percent inhibition of 150 µM AITC-evoked currents in *Xenopus* oocytes expressing WT (black) or WT and R919* (pink) hTRPA1 with 10 µM A-967079 (see Fig. 3a-b). Data represent mean ± SEM. n=9 oocytes per condition. n.s. not significant, two-tailed Student's t-test. (e-f) Ratiometric calcium imaging of HEK293T cells transfected with WT (e) or WT and R919* hTRPA1 (f) pre-treated with either 500 µM or 1 mM carbamazepine. Channels were activated with AITC (100 µM). Images are representatives from three independent experiments. (g-h) Quantification of antagonist-mediated inhibition of 100 µM AITC-evoked changes in Fura-2 ratio in cells from F and G expressing WT (g, black) or WT and R919* (h, pink) hTRPA1. Data represent mean ± SEM of percentage of inhibition of the AITC-evoked maximum response for both transfection types. n = 3 independent experiments, n ≥ 90 cells per condition per experiment. ****, $p<0.0001$, n.s. not significant. one-way ANOVA with Tukey's *post hoc* analysis. (a, b, e, and f) Scale bars indicate 100 µm.

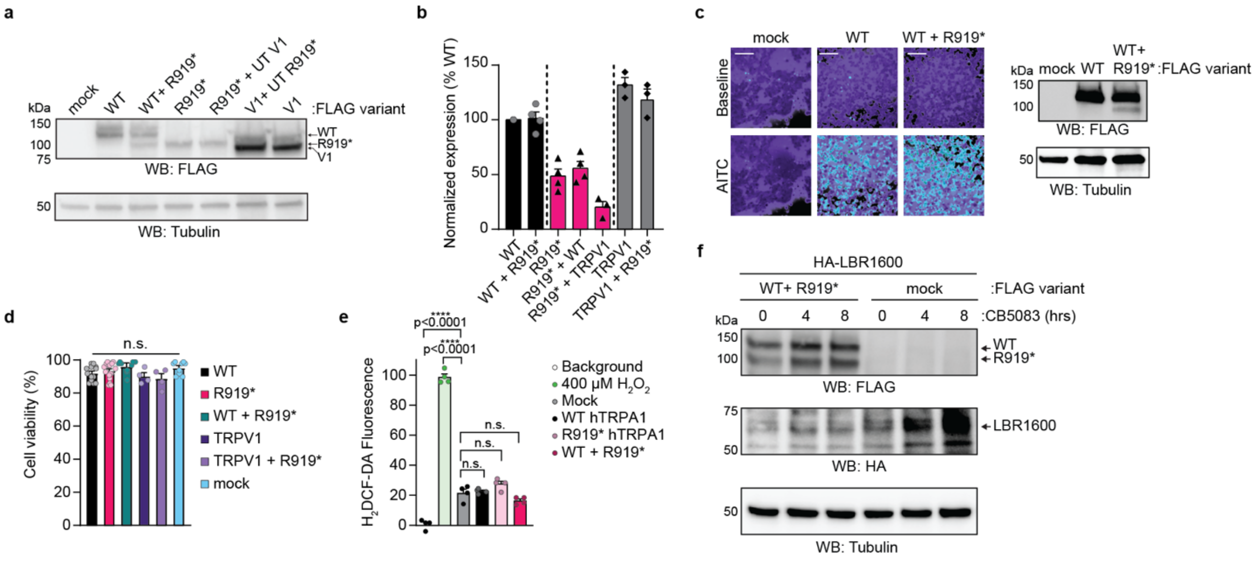

Supplementary Figure 4. The R919* mutant does not affect WT hTRPA1 or TRPV1 expression levels and does not cause general cell stress. (a) Western blot of lysates from transiently transfected HEK293T cells expressing empty vector (mock), 3xFLAG-WT hTRPA1, 3xFLAG-WT and R919* hTRPA1, 3xFLAG-R919* hTRPA1, 3xFLAG-R919* hTRPA1 and untagged (UT) hTRPV1, 3xFLAG-hTRPV1 and untagged (UT) R919* hTRPA1, or 3xFLAG-hTRPV1. Lysates were probed using HRP-conjugated anti-FLAG antibody. Tubulin was the loading control. Blot is representative of three independent experiments. (b) Quantitative analysis of Tubulin-normalized 3xFLAG-tagged hTRPA1 variants or hTRPV1 from (a) relative to WT hTRPA1. Data represent mean ± SEM. n=3 (TRPV1-expressing samples) or 4 (all other samples) independent experiments. Colors indicate WT hTRPA1 (black), R919* (pink) hTRPA1, or WT hTRPV1 (grey) expression in different transfection conditions. (c) Ratiometric calcium imaging (left) and Western blot analysis (right) of HEK293T cells transiently transfected with empty vector (mock), 3xFLAG-WT hTRPA1, or 3xFLAG-WT and R919* hTRPA1. Cells were stimulated with AITC (10 μM). Images are representatives from three independent experiments. Scale bars indicate 100 μm. Lysates were probed using HRP-conjugated anti-FLAG antibody. Tubulin was the loading control. (d) Cell viability of HEK293T cells transiently transfected with the indicated hTRPA1 variants or hTRPV1 was quantified by trypan blue exclusion. Data represent mean ± SEM. n=4 (TRPV1 (dark purple) and TRPV1 with R919* (light purple)), 9 (mock (blue) and WT with R919* (green)), or 17 (WT (black) and R919* (pink)) independent experiments, one-way ANOVA with Bonferroni's *post hoc* analysis. (e) ROS-detection assay to test for oxidative stress. HEK293T cells transfected with empty vector (mock, grey), WT (black), R919* (light pink), or WT and R919* (deep pink) hTRPA1 were loaded with 50 μM H_2DCF-DA dye to detect ROS. Background readings were from buffer only (white). Buffer treated with 400 μM H_2O_2 was the positive control (green) and data were normalized to H_2O_2-evoked H_2DCF-DA fluorescence. Data represent mean ± SEM. n=4 independent experiments, ****, p<0.0001, n.s. not significant. one-way ANOVA with Tukey's *post hoc* analysis. (f) LBR1600 stabilization assay to test for ERAD. To induce ERAD, HEK293T cells co-expressing HA-LBR1600 with empty vector (mock) or 3xFLAG-WT and 3xFLAG R919* hTRPA1 were treated with 10 μM of p97 inhibitor CB5983 for 4 or 8 hrs. Lysates were probed using HRP-conjugated anti-FLAG antibody for TRPA1 or HRP-conjugated anti-HA antibody for LBR1600. Tubulin was the loading control. Blots are representative of three independent experiments. (a, c, and f) Full blots are included in Supplementary Fig. 16.

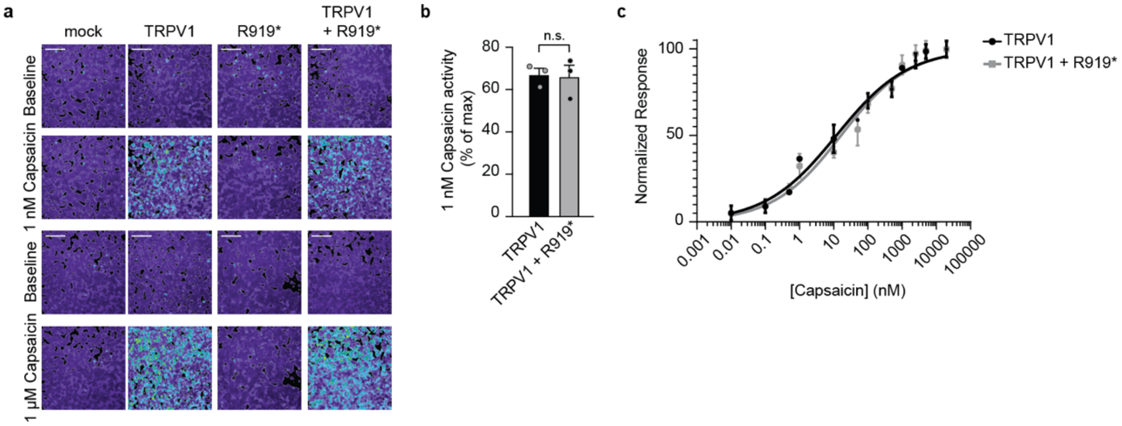

Supplementary Figure 5. The R919* mutant does not affect TRPV1 channel activity. (a) Ratiometric calcium imaging of HEK293T cells transiently transfected with empty vector (mock), hTRPV1, R919* hTRPA1, or hTRPV1 and R919* hTRPA1. Cells were stimulated with 1 nM Capsaicin (top) or 1 µM Capsaicin (bottom). Images are representatives from three independent experiments. Scale bars indicate 100 µm. (b) Quantification of 1 nM Capsaicin-evoked change in Fura-2 ratio relative to maximum response of each expression condition at 1 µM Capsaicin for cells expressing WT hTRPV1 alone (black) or with R919* hTRPA1 (grey). Data represent mean ± SEM. n.s., not significant. n = 3 independent experiments, n ≥ 90 cells per transfection condition per experiment, two-tailed Student's t-test. (c) Dose-response curve of Capsaicin-evoked calcium responses for HEK293T cells transiently transfected with hTRPV1 (black) or hTRPV1 and R919* hTRPA1 (grey). Calcium responses normalized to maximum calcium response at 20 µM Capsaicin. Traces represent the average ± SEM of normalized calcium responses from 3 independent experiments, n = 30 cells per agonist concentration per experiment. Data were fit to a non-linear regression. EC_{50} (95% CI) values are 12.1 nM for hTRPV1 (95% CI: 7.3-19.7 nM) and 16.5 nM for hTRPV1 and R919* hTRPA1 (95% CI: 9.3-28.6 nM).

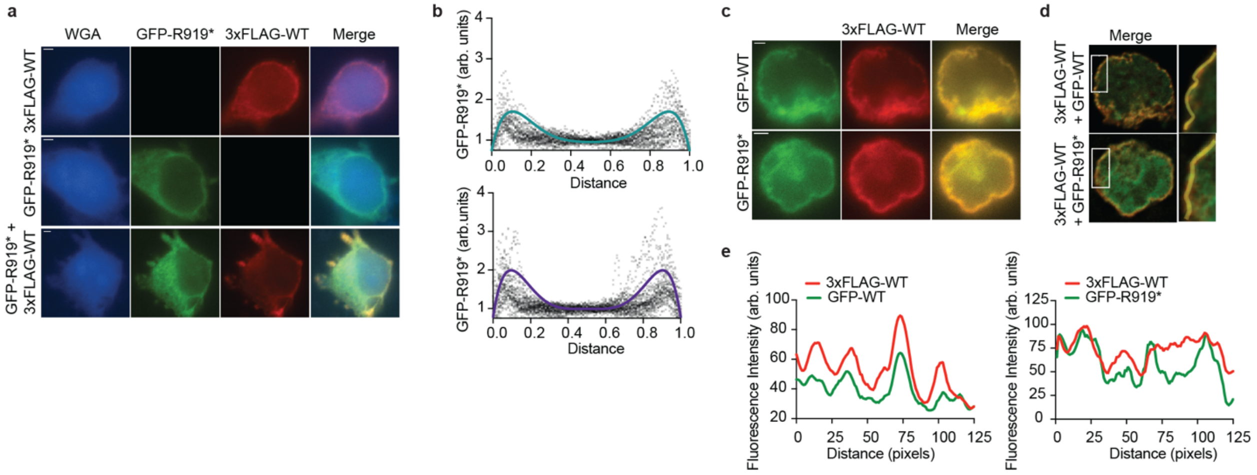

Supplementary Figure 6. Representative immunofluorescence imaging data. (a) Representative raw immunofluorescence images of HEK293T cells transiently transfected with GFP-R919* hTRPA1, 3xFLAG-WT hTRPA1, or GFP-R919* hTRPA1 and 3xFLAG-WT hTRPA1. Cells were stained with anti-GFP (green) and anti-FLAG (red) antibodies. Plasma membrane was labeled with wheat germ agglutinin (blue). Scale bar indicates 2 µm. Images are representative of 3 independent experiments. (b) Combined line scans of HEK293T cell cross-sections with transient transfection of GFP-R919* hTRPA1 (left) or co-transfection of GFP-R919* hTRPA1 and 3xFLAG-WT hTRPA1 (right). Distance and GFP-R919* hTRPA1 fluorescence are normalized relative to total cell width and internal intensity. Representative polynomial lines are overlayed in teal (left) or purple (right). n=30 cells. Arbitrary units, arb. units. (c) Raw images of transiently transfected HEK293T cells used for fluorescence correlation in Fig. 4e. Cells were transiently transfected with 3xFLAG-WT hTRPA1 and GFP-WT hTRPA1 or GFP-R919* hTRPA1, then stained with anti-GFP (green) and anti-FLAG (red) antibodies. Scale bar indicates 2 µm. Images are representative of 3 independent experiments. (d) Deconvolved images of HEK293T cells depicted in (c). Segments of plasma membrane used are magnified (right) for line-scan analysis of red and green signal intensity. Images are representative of 3 independent experiments. (e) Line scans of plasma membrane segments indicated in (d). Arbitrary units, arb. units.

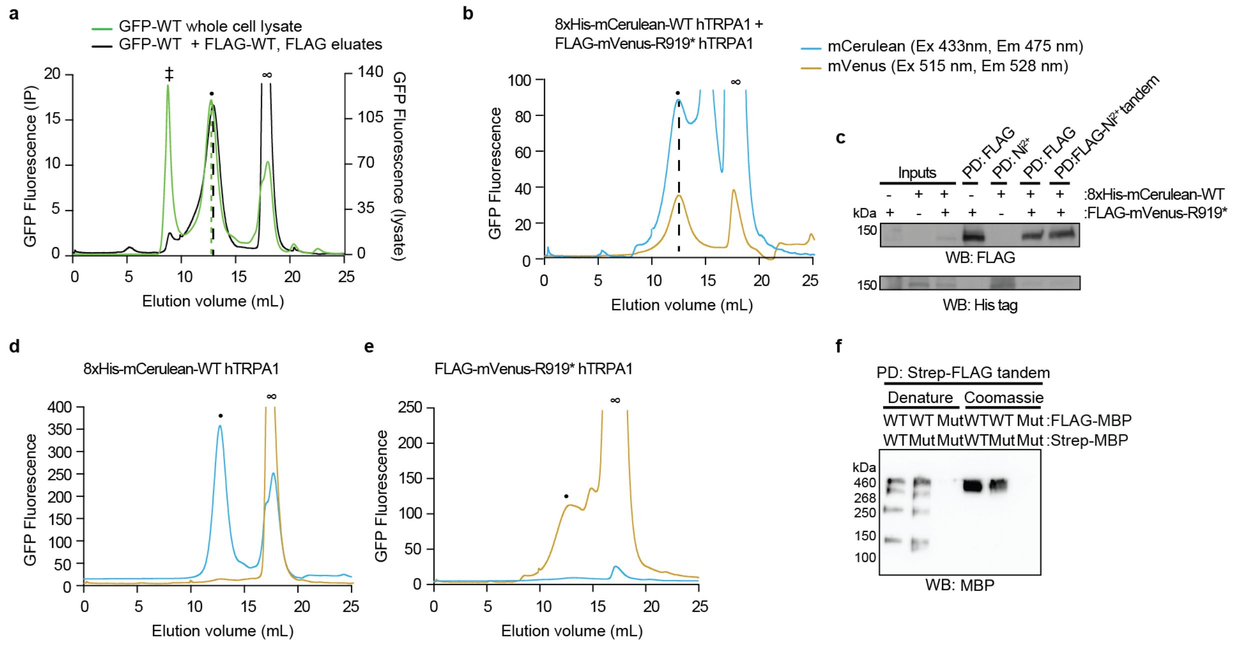

Supplementary Figure 7. FSEC analysis and Native Blue PAGE of WT and WT-R919* TRPA1 complexes. (a) FSEC chromatograms of whole cell lysate from HEK293T cells transiently transfected with GFP-WT hTRPA1 (green trace) or FLAG immunoprecipitated eluates from HEK293T cells transiently co-transfected with GFP-WT hTRPA1 and 3xFLAG-WT hTRPA1 (black trace). Peaks corresponding to void (double cross), tetrameric WT hTRPA1 channels (black dot) and free GFP (infinity symbol) are indicated. Dashed lines denote the center elution volume of each TRPA1 peak. (b) Two-color FSEC chromatogram and (c) Western blot analysis of His-FLAG tandem purified complexes from lysates of HEK293T cells co-expressing 8xHis-mCerulean-WT hTRPA1 and FLAG-mVenus-R919* hTRPA1. Tandem purified eluates were split in two and analyzed at the mCerulean (blue trace) and mVenus (yellow trace) wavelengths. Peaks corresponding to tetrameric hTRPA1 channels (black dot) and free FLAG peptide in elution buffer (infinity symbol) are indicated. Dashed lines denote the center elution volume of each TRPA1 peak. Samples were also analyzed by Western blot (c) using HRP-conjugated anti-FLAG antibody and HRP-conjugated His probe. (d and e) FSEC chromatograms of whole cell lysates from HEK293T cells transiently transfected with 8xHis-mCerulean-WT hTRPA1 (d) or FLAG-mVenus-R919* hTRPA1 (e) analyzed at the mCerulean (blue traces) and mVenus (yellow traces) wavelengths. Peaks corresponding to tetrameric hTRPA1 channels (black dot) and free fluorescent protein (infinity symbol) are indicated. No spectral contamination was observed between mCerulean and mVenus. (a, b, d, and e) Chromatograms are representative of three independent trials. (f) Immunoblotting analysis of tandem-purified WT/WT, WT/R919*, and R919*/R919* hTRPA1 complexes. The indicated FLAG-MBP-tagged and Strep-MBP-tagged constructs were transiently transfected in HEK293T cells. Lysates were tandem purified for Strep- then FLAG-tagged proteins. Eluents were run on a Blue Native PAGE gel after full (left) or partial (right) denaturation. MBP-tagged proteins of tandem purification eluents were probed using anti-MBP antibody. Data representative of 2 independent experiments. (c and f) Full blots are included in Supplementary Fig. 16.

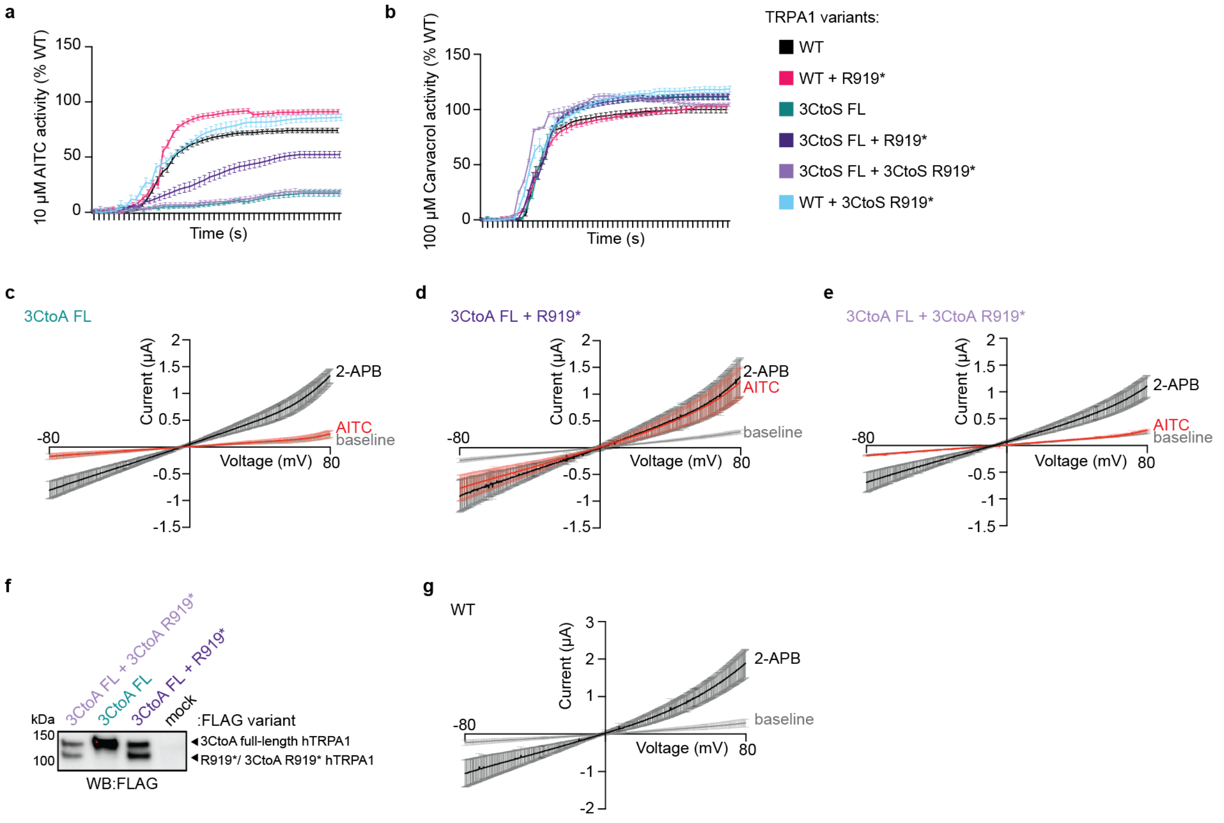

Supplementary Figure 8. R919* TRPA1 subunits directly contribute to functional channels. (a-b) Ratiometric calcium imaging traces of HEK293T cells transiently transfected with WT hTRPA1 (black), 3CtoS FL hTRPA1 (green), WT and R919* hTRPA1 (pink), 3CtoS FL and R919* hTRPA1 (deep purple), 3CtoS FL and 3CtoS R919* hTRPA1 (light purple), or WT and 3CtoS R919* hTRPA1 (blue) from data quantified in Fig. 6b. Cells were stimulated with 10 µM AITC (a) or 100 µM Carvacrol (b). Traces are averages ± SEM of one representative experiment (n = 30 cells) per condition. Data further normalized to WT hTRPA1 response. (c-e) Average I-V relationships from *Xenopus* oocytes presented in Fig. 6c-e expressing 3CtoA hTRPA1 (c, n=7), 3CtoA FL and R919* hTRPA1 (d, n=8), or 3CtoA FL and 3CtoA R919* hTRPA1 (e, n=7) showing baseline currents (grey) and sequentially activated with 150 µM AITC (red) followed by 500 µM 2-APB (black) in the same oocytes. Data represent mean ± SEM. (f) Western blot of lysates from representative oocytes used for recordings in Fig. 6c-e. Lysates were probed using HRP-conjugated anti-FLAG antibody. Blot is representative of one oocyte per injection type (3CtoA hTRPA1 (n=7), 3CtoA FL and R919* hTRPA1 (n=8), 3CtoA FL and 3CtoA R919* hTRPA1 (n=7), or mock (n=7)). Full blot is included in Supplementary Fig. 17. (g) Average I-V relationships from *Xenopus* oocytes expressing WT hTRPA1 showing baseline currents (grey) and those evoked by 500 µM 2-APB (black) in the same oocytes. Data represent mean ± SEM, n=3 independent oocytes. (c-e and g) Extracellular solution contained no calcium.

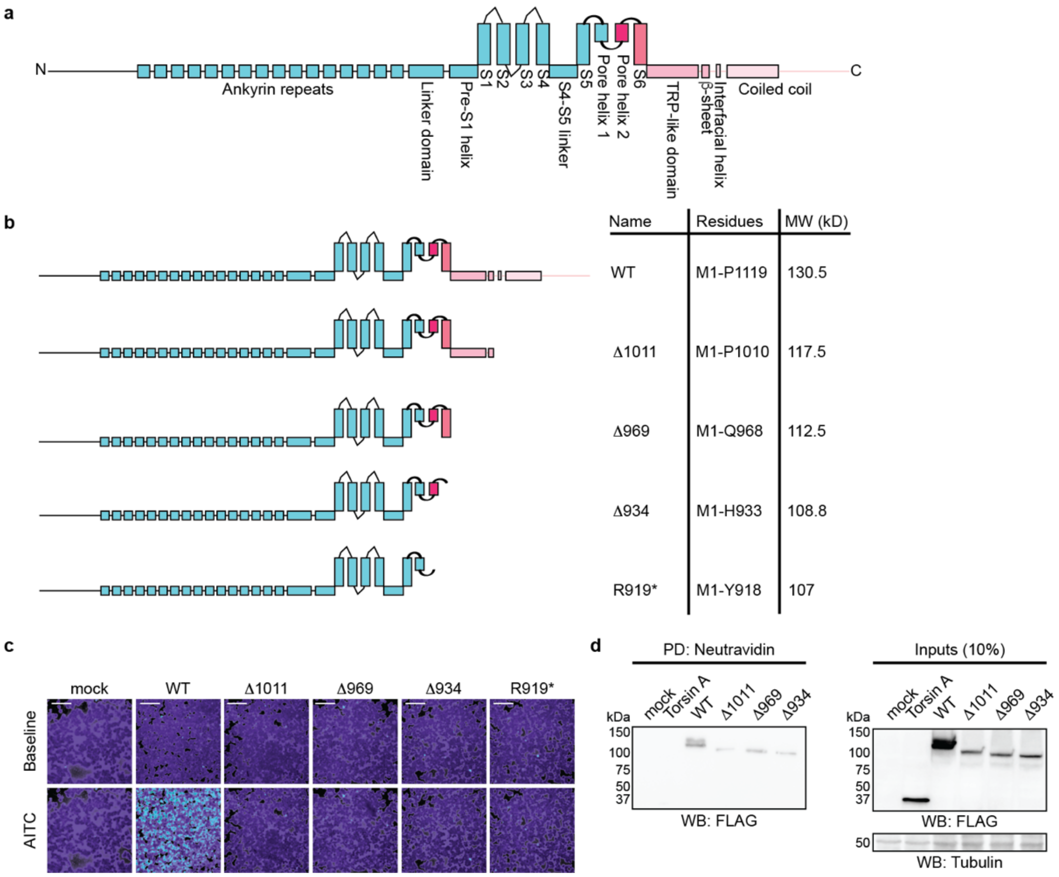

Supplementary Figure 9. Design, activity, expression, and surface localization of C-terminal TRPA1 truncation constructs. (a) Linear diagram depicting major structural domains in a WT hTRPA1 monomer. Colors as indicated in Fig. 7a. (b) Schematic representation and summary of composition of C-terminal TRPA1 truncation mutants assayed in Fig. 7b and c. (c) Ratiometric calcium imaging of HEK293T cells transiently transfected with empty vector (mock) or the indicated 3xFLAG-tagged hTRPA1 constructs. Cells were stimulated with AITC (100 μM, n=60 cells per transfection condition). Scale bars indicate 100 μm. Images representative of three independent experiments. (d) Immunoblotting analysis of 3xFLAG-tagged hTRPA1 constructs or FLAG-Torsin A protein expression in biotin-labeled plasma membranes from transiently transfected HEK293T cells. Biotinylated proteins were precipitated by Neutravidin resin pulldown and probed using HRP-conjugated anti-FLAG antibody. Tubulin from whole cell lysates (10%, inputs) was the loading control. Torsin A was the negative control for plasma membrane localization. Data is representative of three independent experiments. Full blots are included in Supplementary Fig. 17.

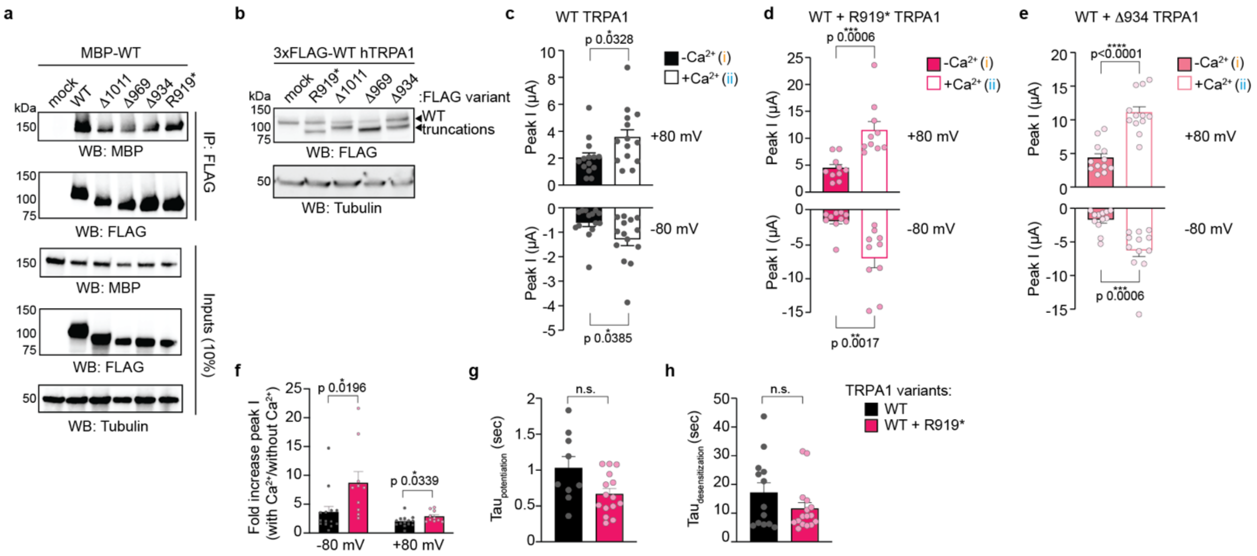

Supplementary Figure 10. Mechanistic dissection of R919*-associated TRPA1 mutant-conferred channel hyperactivity. (a) Immunoblotting analysis of MBP-WT hTRPA1 protein after FLAG immunoprecipitation from lysates of cells co-transfected with empty vector (mock) or 3xFLAG-WT, R919*, or the indicated hTRPA1 C-terminal truncations. Samples were probed using anti-MBP antibody or HRP-conjugated anti-FLAG antibody. Tubulin from whole cell lysates was the loading control. Data is representative of 4 independent experiments. (b) Western blot of lysates from transiently transfected HEK293T cells expressing 3xFLAG-tagged hTRPA1 variants from Fig. 7b, probed using HRP-conjugated anti-FLAG antibody. Tubulin was the loading control. (a-b) Full blots are included in Supplementary Fig. 17. (c-e) Internal analysis of quantified data from Fig. 8d comparing changes to peak current amplitudes at -80 or +80 mV holding potentials in the absence (filled) and presence (open) of 1.8 mM extracellular calcium for WT hTRPA1 (c, black, n=14 independent oocytes), WT and R919* hTRPA1 (d, deep pink, n=10 independent oocytes), or WT and Δ934-1119 hTRPA1 (e, pink, n=12 independent oocytes). Data represent mean ± SEM. ****p<0.0001, ***p<0.001, **p<0.01,*p<0.05. Two-tailed Student's t-test. (f) Quantified degree of potentiation from data in (c) and (d) calculated as -fold increase in the current at -80 or +80 mV after calcium introduction. Data represent mean ± SEM. *p<0.05. n as indicated in c-d, two-tailed Student's t-test. (g-h) Calculated time constants of potentiation (g) and desensitization (h) from fitting data from Fig. 8a and b to a single-exponential function. Data represent mean ± SEM. n.s. not significant. n= 9 (WT) or 15 (WT and R919*) independent oocytes per condition, two-tailed Student's t-test. (f-h) Colors indicate analyses from oocytes expressing WT (black) or WT and R919* (deep pink) hTRPA1.

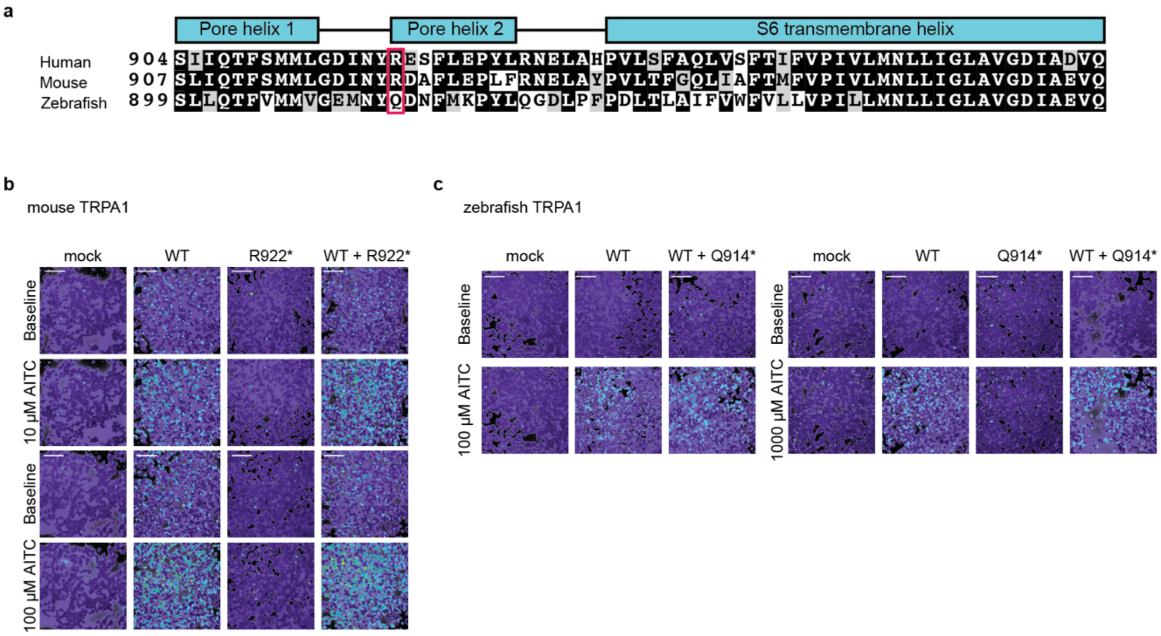

Supplementary Figure 11. Evolutionary conservation of CRAMPT-associated TRPA1 mutant-conferred channel hyperactivity in TRPA1 species orthologues. (a) Alignment of mouse TRPA1 and zebrafish TRPA1 isoform a (zTRPA1a) with the human TRPA1 protein with protein topology indicated above. Amino acid residues are highlighted in black when present in at least two proteins with a similar residue in the third. The human R919 residue is indicated with a pink box. Alignment was built with T-Coffee[6] and BOXSHADE. (b) Representative ratiometric calcium imaging of HEK293T cells transiently transfected with empty vector (mock), WT mTRPA1, R922* mTRPA1, or WT and R922* mTRPA1 from data quantified in Fig. 9a. Cells were stimulated with 10 μM (top) or 100 μM (bottom) AITC. n ≥ 90 cells per concentration. Images representative of four independent experiments. (c) Representative ratiometric calcium imaging of HEK293T cells transiently transfected with empty vector (mock), WT zTRPA1a, Q914* zTRPA1a, or WT and Q914* zTRPA1a from data quantified in Fig. 9b. Cells were stimulated with 100 μM (left) or 1000 μM (right) AITC. n ≥ 90 cells per concentration. Images representative of four independent experiments. (b and c) Scale bars indicate 100 μm.

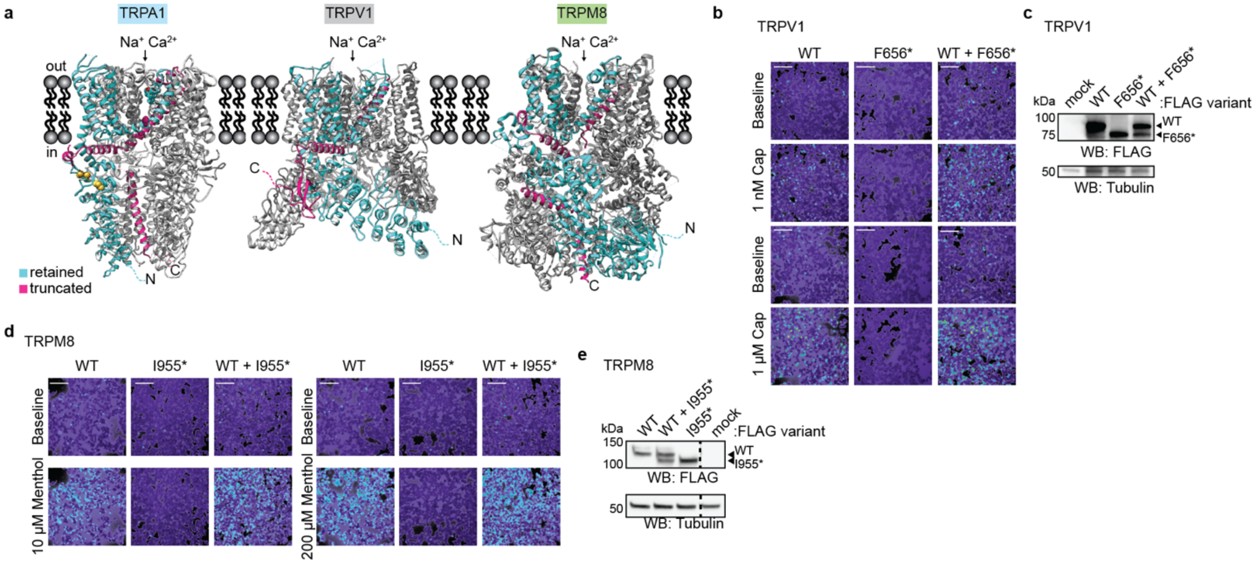

Supplementary Figure 12. A TRPV1 mutant, but not a TRPM8 mutant, lacking the S6 transmembrane helix and cytoplasmic C-terminus confers channel hyperactivity with WT protein. (a) Ribbon diagrams of TRPA1, TRPV1, and TRPM8. Regions retained in R919* hTRPA1, and the S6 and cytoplasmic C-terminus truncations for TRPV1 and TRPM8 are indicated in teal. Regions truncated in these mutants are indicated in pink. Only one subunit is colored for clarity. Models built with the human TRPA1 (PDB: 6V9W), rat TRPV1 (PDB: 7LP9), and *Parus major* TRPM8 (6O6A) Cryo-EM structures in UCSF Chimera. (b) Ratiometric calcium imaging of HEK293T cells transiently transfected with empty vector (mock), 3xFLAG-WT human TRPV1, 3xFLAG-F656* human TRPV1, or 3xFLAG-WT and F656* human TRPV1 from data quantified in Fig. 9c. Cells were stimulated with 1 nM (left) or 1 µM (right) Capsaicin. n ≥ 90 cells per condition. Images are representative of three independent experiments. (c) Western blot of lysates from transiently transfected HEK293T cells from (b), probed using HRP-conjugated anti-FLAG antibody. Tubulin was the loading control. Blot is representative of three independent experiments. (d) Ratiometric calcium imaging of HEK293T cells transiently transfected with empty vector (mock), 3xFLAG-WT rat TRPM8, 3xFLAG-I955* rat TRPM8, or 3xFLAG-WT and I955* rat TRPM8 from data quantified in Fig. 9d. Cells were stimulated with 10 µM (left) or 200 µM (right) Menthol. n ≥ 90 cells per condition. Images are representative of three independent experiments. (e) Western blot of lysates from transiently transfected HEK293T cells from (d), probed using HRP-conjugated anti-FLAG antibody. Tubulin was the loading control. Blot is representative of three independent experiments. (b and d) Scale bars indicate 100 µm. (c and e) Full blots are included in Supplementary Fig. 17.

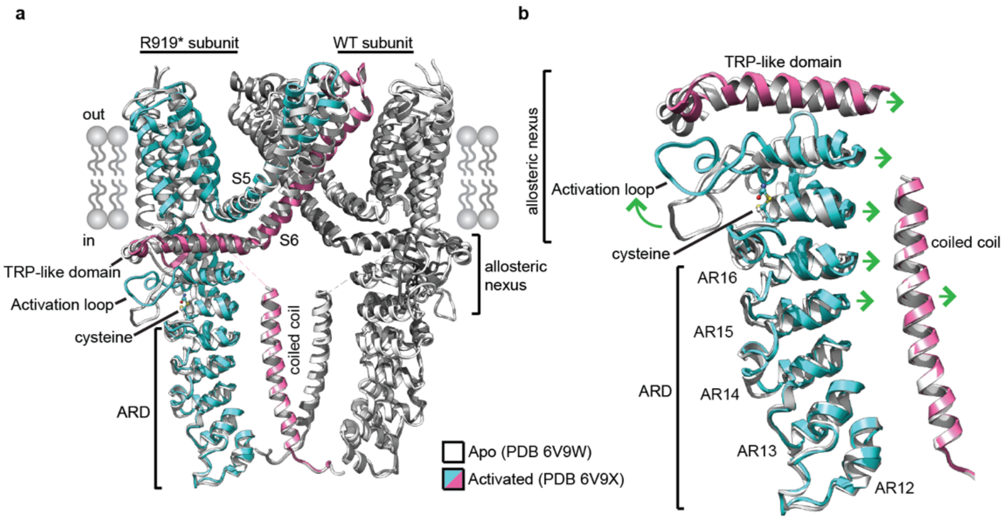

Supplementary Figure 13. Conformational changes associated with TRPA1 activation. (a) Overlay of ribbon diagrams of opposing WT TRPA1 subunits in the closed (Apo, PDB 6V9W, white) and activated (PDB 6V9X, blue/pink or dark gray) states, respectively. The subunit on the left is colored to indicate regions retained in R919* hTRPA1 (teal) and those truncated in this mutant (pink). The subunit on the right (dark grey) is colored to indicate an associated WT TRPA1 subunit. Only two opposing subunits are shown for clarity. Models built with the indicated TRPA1 Cryo-EM structures in UCSF Chimera. (b) Overlay of ribbon diagrams of the TRPA1 allosteric nexus, membrane-proximal ankyrin repeat domain (ARD), and coiled coil colored as in (a). Green arrows indicate regions and direction of gating associated conformational changes.

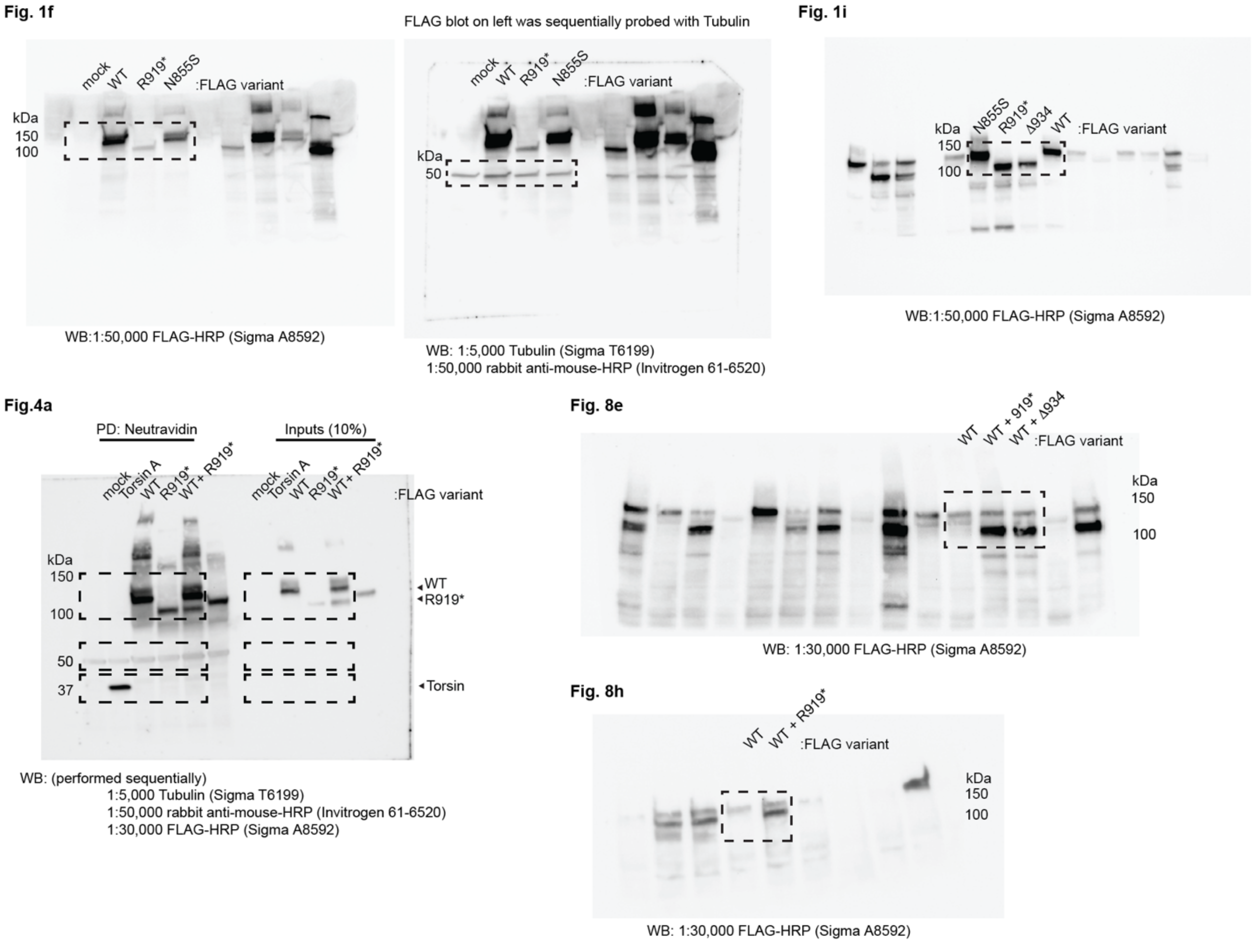

Supplementary Figure 14. Uncut full Western blots shown in Fig. 1f and i, Fig. 4a, and Fig 8e and h. The regions surrounded by dashed lines represent the panels in the respective figures.

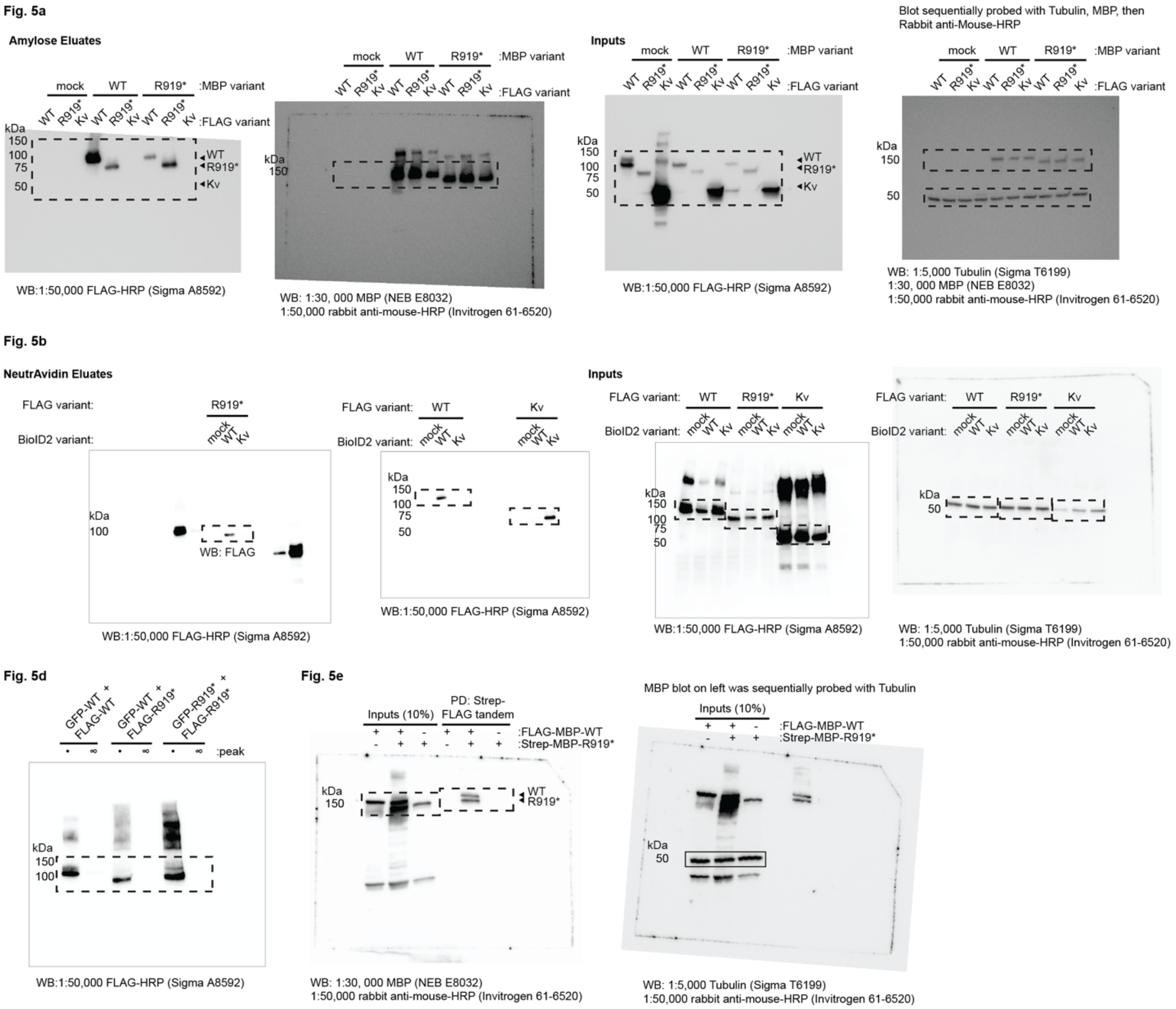

Supplementary Figure 15. Uncut full Western blots shown in Fig. 5a, b, d, and e. The regions surrounded by dashed lines represent the panels in the respective figures.

Supplementary Figure 1b

Blot sequentially probed with Tubulin, Rabbit anti-Mouse-HRP, then FLAG-HRP

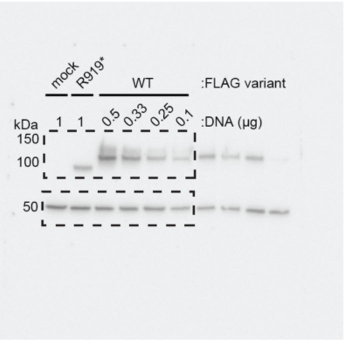

WB: 1:5,000 Tubulin (Sigma T6199)
1:50,000 rabbit anti-mouse-HRP (Invitrogen 61-6520)
1:50,000 FLAG-HRP (Sigma A8592)

Supplementary Figure 4a

Blot sequentially probed with Tubulin, Rabbit anti-Mouse-HRP, then FLAG-HRP

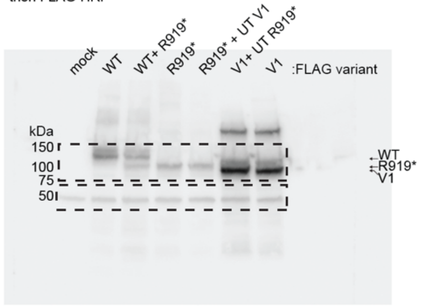

WB: 1:5,000 Tubulin (Sigma T6199)
1:50,000 rabbit anti-mouse-HRP (Invitrogen 61-6520)
1:50,000 FLAG-HRP (Sigma A8592)

Supplementary Figure 4c

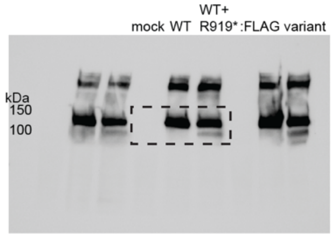

WB:1:50,000 FLAG-HRP (Sigma A8592)

FLAG blot above was sequentially probed with Tubulin

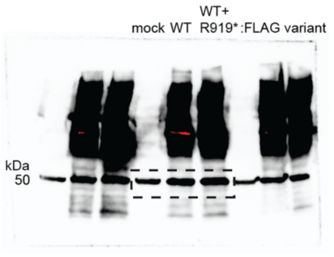

WB: 1:5,000 Tubulin (Sigma T6199)
1:50,000 rabbit anti-mouse-HRP (Invitrogen 61-6520)

Supplementary Figure 4f

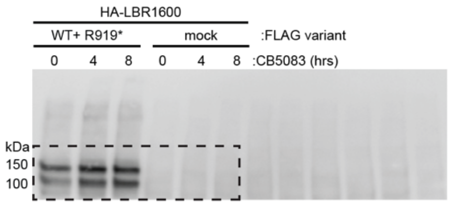

WB:1:30,000 FLAG-HRP (Sigma A8592)

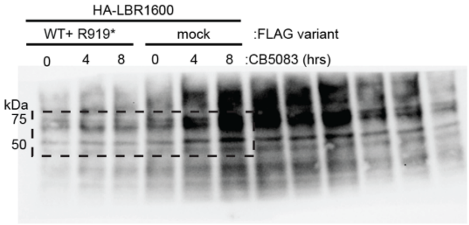

WB:1:2,000 HA-HRP (Roche 12013819001)

HA blot on left was stripped and sequentially probed with Tubulin

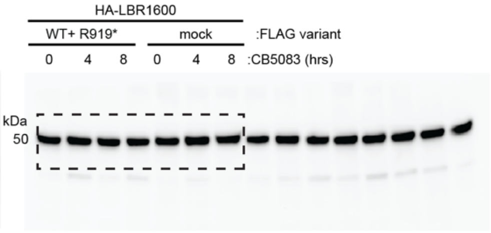

WB: 1:5,000 Tubulin (Sigma T6199)
1:50,000 rabbit anti-mouse-HRP (Invitrogen 61-6520)

Supplementary Figure 7c

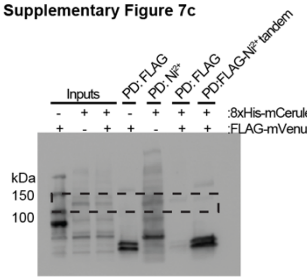

WB:1:50,000 His-Probe-HRP (Thermo 15165)

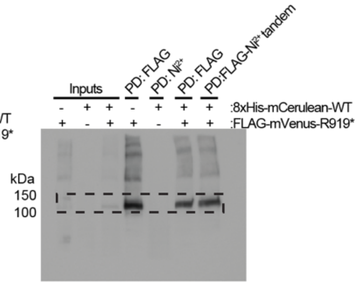

WB:1:50,000 FLAG-HRP (Sigma A8592)
His blot on left was stripped and sequentially probed with FLAG

Supplementary Figure 7f

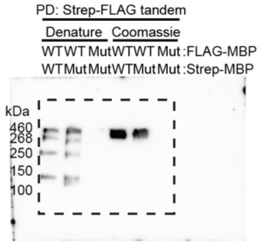

WB: 1:30, 000 MBP (NEB E8032)
1:50,000 rabbit anti-mouse-HRP (Invitrogen 61-6520)

Supplementary Figure 16. Uncut full Western blots shown in Supplementary Fig. 1b, 4a, 4c, 4f, 7c, and 7f. The regions surrounded by dashed lines represent the panels in the respective figures.

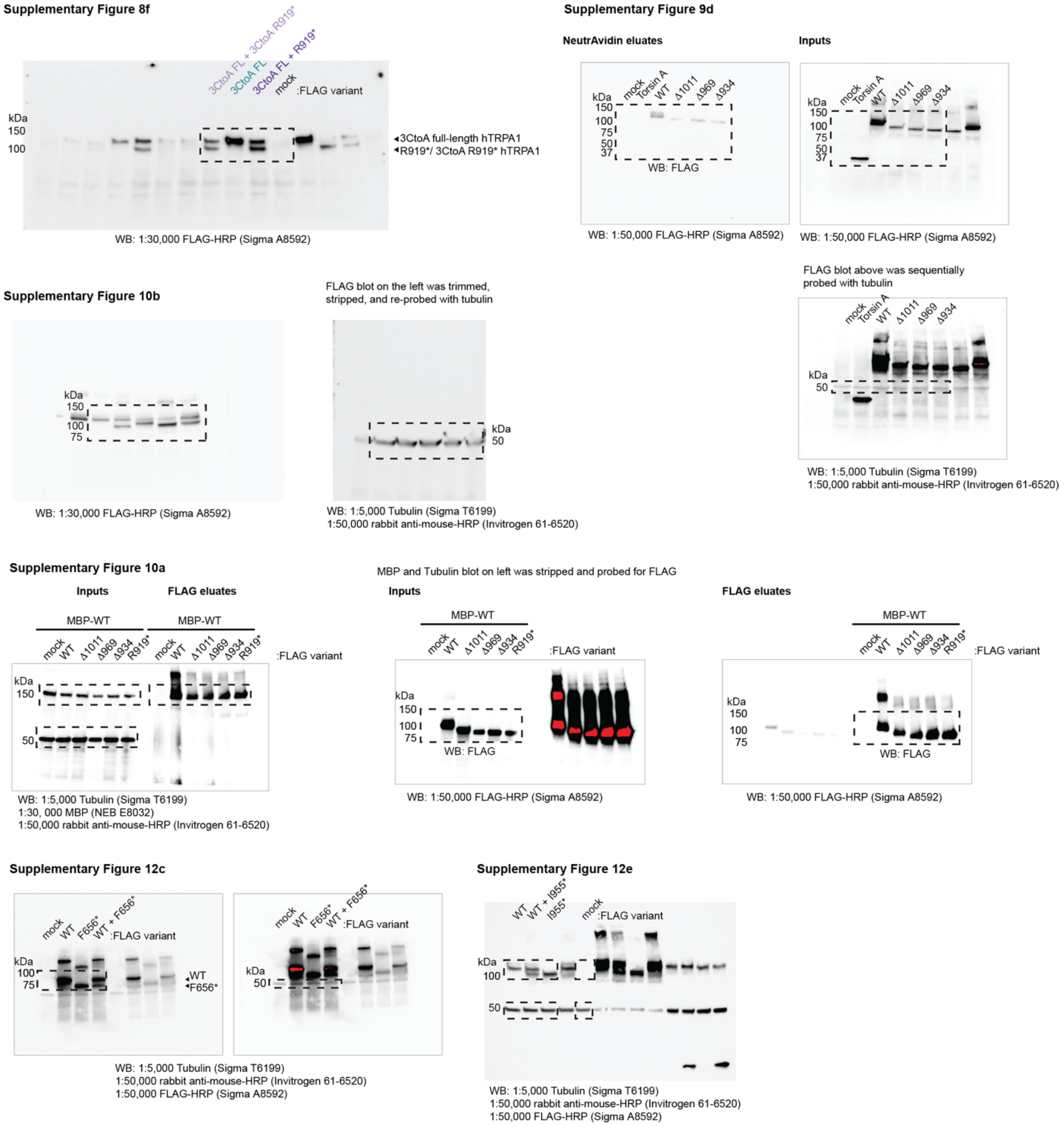

Supplementary Figure 17. Uncut full Western blots shown in Supplementary Fig. 8f, 9d 10b, 10a, 12c, and 12e. The regions surrounded by dashed lines represent the panels in the respective figures.

Supplementary Information References

1 Chang, A. *et al.* A Calmodulin C-Lobe Ca(2+)-Dependent Switch Governs Kv7 Channel Function. *Neuron* **97**, 836-852 e836, (2018).

2 Paulsen, C. E. *et al.* Peroxide-dependent sulfenylation of the EGFR catalytic site enhances kinase activity. *Nat Chem Biol* **8**, 57-64, (2011).

3 Tsai, P. L., Zhao, C. & Schlieker, C. Methodologies to monitor protein turnover at the inner nuclear membrane. *Methods Enzymol* **619**, 47-69, (2019).

4 Tsai, P. L., Zhao, C., Turner, E. & Schlieker, C. The Lamin B receptor is essential for cholesterol synthesis and perturbed by disease-causing mutations. *Elife* **5**, (2016).

5 Paulsen, C. E., Armache, J. P., Gao, Y., Cheng, Y. & Julius, D. Structure of the TRPA1 ion channel suggests regulatory mechanisms. *Nature* **520**, 511-517, (2015).

6 Notredame, C., Higgins, D. G. & Heringa, J. T-Coffee: A novel method for fast and accurate multiple sequence alignment. *J Mol Biol* **302**, 205-217, (2000).
