# Supplementary material for: Molecular mechanism of hyperactivation conferred by a truncation of TRPA1
Source: Nat Commun. 2023 May 19;14:2867. doi: 10.1038/s41467-023-38542-1 (PMC10199097; doi:10.1038/s41467-023-38542-1)
Supplement: Supplementary file 3 — Reporting Summary [file 41467_2023_38542_MOESM3_ESM.pdf]

nature portfolio

Corresponding author(s): Candice E. Paulsen

Last updated by author(s): Apr 27, 2023

Reporting Summary

Nature Portfolio wishes to improve the reproducibility of the work that we publish. This form provides structure for consistency and transparency in reporting. For further information on Nature Portfolio policies, see our Editorial Policies and the Editorial Policy Checklist.

Statistics

For all statistical analyses, confirm that the following items are present in the figure legend, table legend, main text, or Methods section.

n/a	Confirmed	
☐	☒	The exact sample size (*n*) for each experimental group/condition, given as a discrete number and unit of measurement
☐	☒	A statement on whether measurements were taken from distinct samples or whether the same sample was measured repeatedly
☐	☒	The statistical test(s) used AND whether they are one- or two-sided<br>*Only common tests should be described solely by name; describe more complex techniques in the Methods section.*
☒	☐	A description of all covariates tested
☐	☒	A description of any assumptions or corrections, such as tests of normality and adjustment for multiple comparisons
☐	☒	A full description of the statistical parameters including central tendency (e.g. means) or other basic estimates (e.g. regression coefficient) AND variation (e.g. standard deviation) or associated estimates of uncertainty (e.g. confidence intervals)
☐	☒	For null hypothesis testing, the test statistic (e.g. *F*, *t*, *r*) with confidence intervals, effect sizes, degrees of freedom and *P* value noted<br>*Give P values as exact values whenever suitable.*
☒	☐	For Bayesian analysis, information on the choice of priors and Markov chain Monte Carlo settings
☒	☐	For hierarchical and complex designs, identification of the appropriate level for tests and full reporting of outcomes
☐	☒	Estimates of effect sizes (e.g. Cohen's *d*, Pearson's *r*), indicating how they were calculated

Our web collection on statistics for biologists contains articles on many of the points above.

Software and code

Policy information about availability of computer code

Data collection: Commercial software was used in all cases. Functional data were collected with MetaFluor v7.8.13 and pClamp 11. Size exclusion chromatography data was collected with Unicorn v7.2. Western blots were imaged with a BioRad ChemiDoc imager.

Data analysis: Commercial software or open-source software were used in all cases. Data were analyzed in pClamp 11, Microscoft Excel, or GraphPad Prism. Western blots and single stack immunofluorescence images were analyzed in ImageJ. Immunofluorescence images were deconvolved with Huygens software and further processed in Photoshop CS4.

For manuscripts utilizing custom algorithms or software that are central to the research but not yet described in published literature, software must be made available to editors and reviewers. We strongly encourage code deposition in a community repository (e.g. GitHub). See the Nature Portfolio guidelines for submitting code & software for further information.

Data

Policy information about availability of data

All manuscripts must include a data availability statement. This statement should provide the following information, where applicable:

- Accession codes, unique identifiers, or web links for publicly available datasets
- A description of any restrictions on data availability
- For clinical datasets or third party data, please ensure that the statement adheres to our policy

All data included in the main manuscript figures are available in the accompanying Source Data file. All graphed data in the supplementary figures are available from

the corresponding author upon reasonable request. All full-size Western blots are included in Supplementary Figures 14-17. Details about plasmids and primers used to build constructs used in main manuscript figures are included in the accompanying Source Data file. The following PDB files were used in this study 6V9W (https://www.rcsb.org/structure/6v9w), 7LP9 (https://www.rcsb.org/structure/7LP9), 6O6A (https://www.rcsb.org/structure/6o6a), and 6V9X (https://www.rcsb.org/structure/6V9X).

Human research participants

Policy information about studies involving human research participants and Sex and Gender in Research.

Reporting on sex and gender	N/A
Population characteristics	N/A
Recruitment	N/A
Ethics oversight	N/A

Note that full information on the approval of the study protocol must also be provided in the manuscript.

Field-specific reporting

Please select the one below that is the best fit for your research. If you are not sure, read the appropriate sections before making your selection.

☒ Life sciences ☐ Behavioural & social sciences ☐ Ecological, evolutionary & environmental sciences

For a reference copy of the document with all sections, see nature.com/documents/nr-reporting-summary-flat.pdf

Life sciences study design

All studies must disclose on these points even when the disclosure is negative.

Sample size	No calculations were made to predetermine sample size. Sample sizes were chosen based on literature review and the number of independent experiments required for strong inference of meaningful conclusions. For each biochemical assay (pulldown experiments, FSEC analysis, or surface biotinylation assay), results were confirmed with a minimum of three independent experiments performed with separate cells on separate days. For ratiometric calcium imaging experiments, quantifications based on 30-40 cells is common (Story et al Cell 2003, 112: 819-829) and are usually collected from a single imaging data set. In each of our ratiometric calcium imaging experiments, we quantify a minimum of 30 cells each from three individual imagining data sets (90 cells total) collected on a single day (one replicate) and further increase our rigor by performing a minimum of three independent experiments collected with separate batches of cells and on separate days. For TEVC experiments, it is field standard to collect data from at least 4 oocytes for each condition (Cordero-Morales et al PNAS 2011, 108: E1184-E1191; Velisetty et al Sci Reports 2017, 7: 9861). For all data presented in the main text figures, we collected data from at least 5 oocytes.
Data exclusions	For electrophysiology or ratiometric calcium imaging data, cells expressing wild type or full-length TRPA1 constructs that were unresponsive to any channel agonists were excluded from data analysis. Additionally, for both functional assays, cells with high basal activity (e.g., high Fura-2 ratio or current before agonist addition) were considered pre-activated and excluded from data analysis to ensure we were only quantifying agonist-evoked responses. Otherwise, no data were excluded from analysis for other experiment types.
Replication	All results were successfully replicated; number of independent experiments (i.e., replicates) indicated in the text. Data reproducibility for all biochemical assays and ratiometric calcium imaging experiments was ensured by having replicates for each experiment type independently contributed by two or three co-authors on separate days and from separate batches of transfected HEK293T cells. For electrophysiology work, replicates were performed on at least five (5) oocytes per condition with data collected on different days and from independent batches of Xenopus laevis oocytes. All attempts at replication were successful.
Randomization	Samples were not randomized; it is not technically or practically feasible to do so for the biochemical and functional assays performed in this study.
Blinding	Researchers were not blinded; it was not technically or practically feasible to do so for the biochemical and functional assays performed in this study. Additionally, all measurements in this study are not subjective. For each experiment type, raw data was collected and analyzed.

Reporting for specific materials, systems and methods

We require information from authors about some types of materials, experimental systems and methods used in many studies. Here, indicate whether each material, system or method listed is relevant to your study. If you are not sure if a list item applies to your research, read the appropriate section before selecting a response.

Materials & experimental systems

n/a	Involved in the study
☐	☒ Antibodies
☐	☒ Eukaryotic cell lines
☒	☐ Palaeontology and archaeology
☒	☐ Animals and other organisms
☒	☐ Clinical data
☒	☐ Dual use research of concern

Methods

n/a	Involved in the study
☒	☐ ChIP-seq
☒	☐ Flow cytometry
☒	☐ MRI-based neuroimaging

Antibodies

Antibodies used

Anti-FLAG HRP conjugate (1:30,000, Sigma A8592), Anti-Tubulin (1:5,000, Sigma T6199), Rabbit anti-Mouse HRP conjugate (1:25,000, Thermo 61-6520), anti-MBP (1:30,000, New England Biolabs E8032), anti-Strep HRP conjugate (1:30,000, IBA 2-1509-001), anti-HA HRP conjugate (1:2,000, Roche 12013819001), His probe HRP conjugate (1:50,000, Thermo 15165), anti-FLAG antibody (1:1,000, Sigma F1804), anti-GFP antibody (1:1,000, Thermo G10362), AffiniPure goat anti-mouse IgG AlexaFluor594-conjugated (1:1,000, Jackson ImmunoResearch 115-585), AffiniPure Goat anti-Rabbit IgG AlexaFluor488-conjugated (1:1,000, Jackson ImmunoResearch 111-545)

Validation

All antibodies used in this study are commercially available and have been validated by manufacturer. Any validation statements are available on the manufacturer's website. The RRID# of each antibody is also provided here:
Anti-FLAG HRP conjugate (1:30,000, Sigma A8592), RRID:AB_429702
Anti-Tubulin (1:5,000, Sigma T6199), RRID:AB_477583
Rabbit anti-Mouse HRP conjugate (1:25,000, Thermo 61-6520), RRID:AB_2533933
anti-MBP (1:30,000, New England Biolabs E8032), RRID:AB_1559730
anti-Strep HRP conjugate (1:30,000, IBA 2-1509-001), https://www.iba-lifesciences.com/strepmab-classic-hrp-conjugate/2-1509-001
anti-HA HRP conjugate (1:2,000, Roche 12013819001), RRID:AB_390917
anti-FLAG antibody (1:1,000, Sigma F1804), RRID:AB_262044
anti-GFP antibody (1:1,000, Thermo G10362), RRID:AB_2536526
AffiniPure goat anti-mouse IgG AlexaFluor594-conjugated (1:1,000, Jackson ImmunoResearch 115-585), RRID:AB_2338871
AffiniPure Goat anti-Rabbit IgG AlexaFluor488-conjugated (1:1,000, Jackson ImmunoResearch 111-545), RRID:AB_2338052

Eukaryotic cell lines

Policy information about cell lines and Sex and Gender in Research

Cell line source(s)	HEK293T cells were purchased directly from ATCC (CRL-3216).
Authentication	Authenticated by vendor, ATCC (CRL-3216).
Mycoplasma contamination	Cell lines used in this study tested negative for mycoplasma contamination.
Commonly misidentified lines (See ICLAC register)	No commonly misidentified cell lines were used in the study.
